# Translating Neurocognitive Models of Auditory-Verbal Hallucinations into Therapy: Using Real-time fMRI-Neurofeedback to Treat Voices

**DOI:** 10.3389/fpsyt.2016.00103

**Published:** 2016-06-27

**Authors:** Thomas Fovet, Natasza Orlov, Miriam Dyck, Paul Allen, Klaus Mathiak, Renaud Jardri

**Affiliations:** ^1^Univ Lille, CNRS, UMR-9193, psyCHIC team & CHU Lille, Psychiatry Dpt (CURE), Fontan Hospital, Lille, France; ^2^Department of Psychosis Studies, Institute of Psychiatry Psychology and Neuroscience, King’s College London, London, UK; ^3^Department of Psychiatry, Psychotherapy and Psychosomatics, JARA-Brain, RWTH Aachen University, Aachen, Germany; ^4^Department of Psychology, University of Roehampton, London, UK

**Keywords:** hallucinations, neurofeedback, fMRI, therapeutic strategies, anterior cingulate cortex

## Abstract

Auditory-verbal hallucinations (AVHs) are frequent and disabling symptoms, which can be refractory to conventional psychopharmacological treatment in more than 25% of the cases. Recent advances in brain imaging allow for a better understanding of the neural underpinnings of AVHs. These findings strengthened transdiagnostic neurocognitive models that characterize these frequent and disabling experiences. At the same time, technical improvements in real-time functional magnetic resonance imaging (fMRI) enabled the development of innovative and non-invasive methods with the potential to relieve psychiatric symptoms, such as fMRI-based neurofeedback (fMRI-NF). During fMRI-NF, brain activity is measured and fed back in real time to the participant in order to help subjects to progressively achieve voluntary control over their own neural activity. Precisely defining the target brain area/network(s) appears critical in fMRI-NF protocols. After reviewing the available neurocognitive models for AVHs, we elaborate on how recent findings in the field may help to develop strong *a priori* strategies for fMRI-NF target localization. The first approach relies on imaging-based “trait markers” (i.e., persistent traits or vulnerability markers that can also be detected in the presymptomatic and remitted phases of AVHs). The goal of such strategies is to target areas that show aberrant activations during AVHs or are known to be involved in compensatory activation (or resilience processes). Brain regions, from which the NF signal is derived, can be based on structural MRI and neurocognitive knowledge, or functional MRI information collected during specific cognitive tasks. Because hallucinations are acute and intrusive symptoms, a second strategy focuses more on “state markers.” In this case, the signal of interest relies on fMRI capture of the neural networks exhibiting increased activity during AVHs occurrences, by means of multivariate pattern recognition methods. The fine-grained activity patterns concomitant to hallucinations can then be fed back to the patients for therapeutic purpose. Considering the potential cost necessary to implement fMRI-NF, proof-of-concept studies are urgently required to define the optimal strategy for application in patients with AVHs. This technique has the potential to establish a new brain imaging-guided psychotherapy for patients that do not respond to conventional treatments and take functional neuroimaging to therapeutic applications.

## Introduction

Auditory-verbal hallucinations (AVHs), i.e., hearing voices in the absence of appropriate external stimuli, are frequent experiences in schizophrenia, with a lifetime prevalence of 60–80% (1, 2). AVHs are often strongly disabling symptoms, which can be refractory to conventional psychopharmacological treatment in more than 25% of the cases (3). A recent meta-analysis supports the effectiveness of cognitive–behavioral therapy (CBT) in the treatment of AVHs (4). However, in the specific case of treatment-refractory symptoms, CBT seems to have modest and only short-term benefits (5, 6).

In recent years, the number of brain imaging studies in the field of AVHs has grown substantially, leading to a better understanding of this subjective phenomenon (7, 8). Recent progress in deciphering the neural underpinnings of AVHs has strengthened transdiagnostic neurocognitive models that characterize AVHs, but, more specifically, these findings built the bases for new therapeutic strategies. Indeed, brain imaging now allows for the identification of therapeutic targets by determining the brain regions involved in the occurrence of AVHs. For example, based on findings implicating the left temporoparietal cortex in AVHs, *repetitive Transcranial Magnetic Stimulation* (rTMS), a non-invasive brain stimulation method, has been used to target this region and shown to have a significant, although moderate, effect in alleviating drug-resistant AVHs (9).

Recently, technical improvements in real-time functional magnetic resonance imaging (fMRI) have enabled the development of fMRI-based neurofeedback (fMRI-NF) (10). During fMRI-NF, brain activity is measured in real time and fed back to the participant, usually using visual or auditory information, in order to facilitate voluntary control over the participant’s own neural activity. Considering the advances in the identification of anatomical and functional changes linked with AVHs, fMRI-NF strategies constitute a promising tool, giving the possibility for patients to normalize their brain activity level or connectivity strength in the AVHs-specific brain regions, and thus reduce symptom severity. Precisely defining the target brain area/network(s) appears crucial for future fMRI-NF protocols designed to treat AVHs.

After briefly reviewing current literature about the neural basis of AVHs (mainly neurocognitive models and brain imaging findings) and providing an overview of how fMRI-NF can be used in psychiatry, the review will then elaborate on how recent advances in the field may help to develop strong *a priori* strategies for fMRI-NF target localization. Three different fMRI-NF strategies dedicated to AVHs’ treatment will be proposed. Current limits, potential difficulties for patients with schizophrenia to benefit from fMRI-NF, as well as future directions will be critically discussed.

## What is fMRI-Neurofeedback?

### fMRI-Neurofeedback: Principles

**Figure 1:**
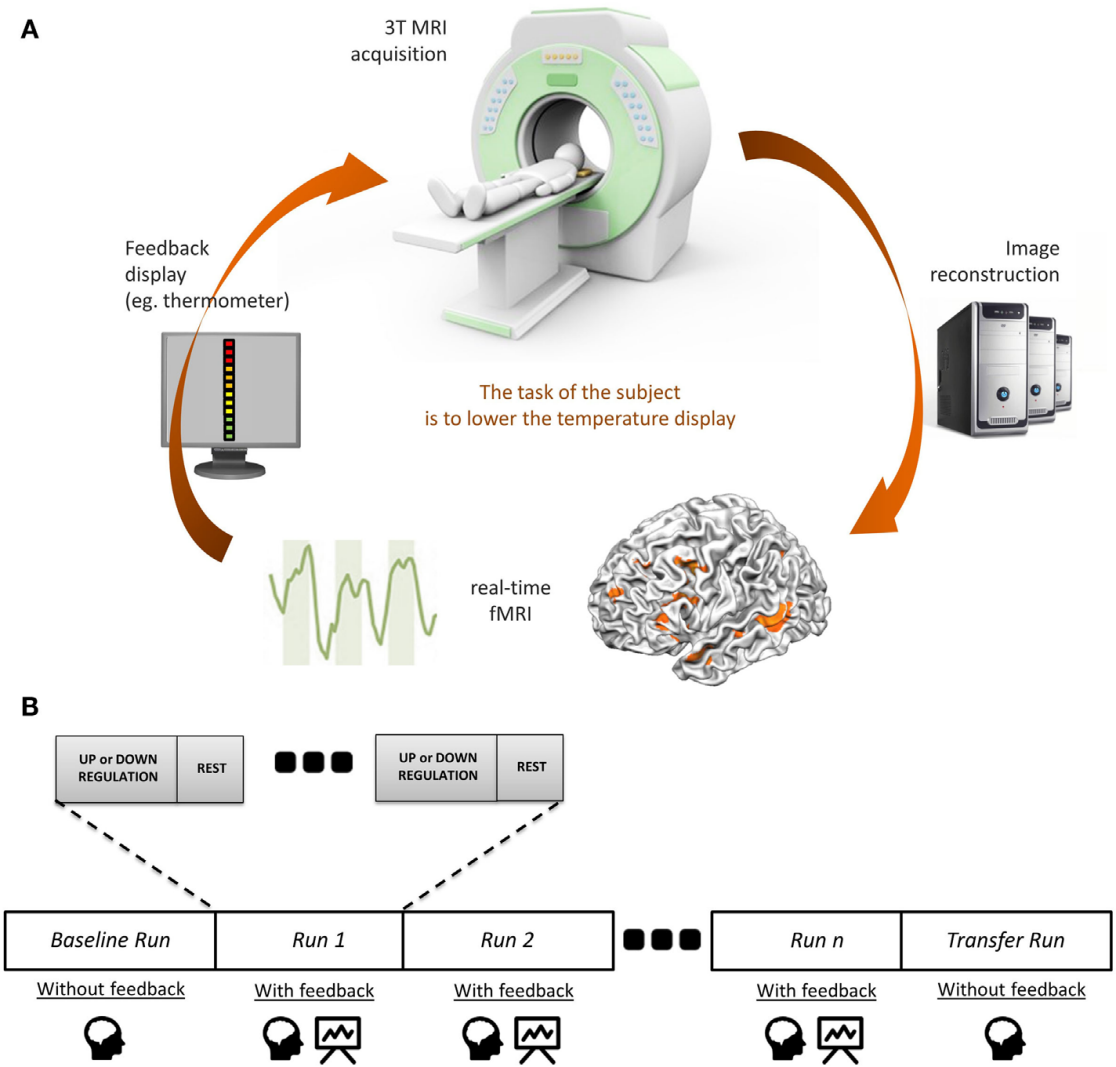
**The principles of fMRI-neurofeedback**. **(A)** Diagram of an fMRI-based neurofeedback system. **(B)** The neurofeedback training in fMRI-neurofeedback protocols.

Neurofeedback is a non-invasive technique enabling participants to achieve voluntary control over the neuronal activity of one or more brain regions [for a recent review on the technique, see Ref. (11)]. In the case of fMRI-NF, this is accomplished by deriving and presenting blood oxygen level-dependent (BOLD) signal derived from the target brain area(s) to the subject in real time (12). Visual feedback is primarily used, but neurofeedback derived from other or combination of different modalities is also possible. Visual feedback can be presented in various formats: from a thermometer display to more complex interfaces [e.g., social feedback Ref. (13)]. The participants use this feedback to self-regulate their neuronal response or adjust their cognitive strategy, during the experimental task in real time (see Figure 1A). They must be informed in detail(s) of the hemodynamic delay of 4 or 5 s (due to the BOLD response) to update the neurofeedback signal. The general experimental design of an fMRI-NF protocol is described in Figure 1B. This technique is currently being used in cognitive modification (14) and clinical trials (15).

### fMRI-Neurofeedback in Psychiatry

fMRI-based neurofeedback could be a useful tool in psychiatry. Numerous studies have shown the benefits of fMRI-NF to relieve non-psychiatric clinical symptoms. Haller et al. demonstrated therapeutic effects of fMRI-NF (focusing on downregulation of auditory cortex) in the treatment of chronic tinnitus (16). deCharms et al. also published promising results for the management of chronic pain (17), although these results failed to be replicated (12).

Furthermore, recent progress in the field of brain imaging has allowed the identification of functional changes associated with a range of psychiatric symptoms (18). Because fMRI-NF can potentially be used to normalize the activity level of specific brain regions (which should be a key issue in new treatments), fMRI-NF could offer a new interesting way to treat mental health symptoms (19). To date, promising positive results have been already demonstrated in major depressive disorder (15, 20) and addiction (21–23).

### Why Using fMRI-Neurofeedback for Auditory-Verbal Hallucinations in Schizophrenia?

In a paper published in 2012, McCarthy-Jones stressed the potential interests of developing neurofeedback as a new treatment of AVHs (24). In the past decade, significant progress in identifying the neural underpinning of AVHs has been made. This knowledge can inform on a target region for fMRI-NF.

To date, only a few studies reporting the use of neurofeedback in patients with schizophrenia have been published. Most of these studies used electroencephalogram (EEG)-based neurofeedback (the principle is the same as fMRI-NF, but the brain activity is measured with EEG) [e.g., Ref. (25–27), with one current study running a trial for the treatment of AVHs (28)]. Two studies have used fMRI-NF in patients with schizophrenia. Ruiz et al. demonstrated that patients with schizophrenia (*n* = 9) were able to achieve voluntary control of bilateral anterior insula cortex using an fMRI-NF protocol (29). Participants completed four training sessions spread over 2 weeks. Each training session comprised three runs of self-regulation training. Each run consisted of six upregulation and seven baseline blocks (30 s blocks). Patients were instructed that the recall of emotionally relevant past experiences combined with the feedback could enable them to control the thermometer bars. No specific emotional cues or recall strategies were given. The gain in the voluntary control was associated with behavioral changes assessed on a facial emotion recognition task (i.e., patients recognized disgust faces more accurately and happy faces less accurately after the fMRI-NF training). Furthermore, the training was associated with an increase in the number of the incoming and outgoing effective connections in the anterior insula. This proof-of-concept study demonstrates that patients with schizophrenia can not only benefit from fMRI-NF and learn volitional brain regulation but also find that such learning is accompanied with behavioral changes and neurophysiological changes in the underlying brain network (29). More recently, Cordes et al. showed that patients with schizophrenia (*n* = 11) were also able to learn to control the activity of their anterior cingulate cortex (ACC) (30). Here, three fMRI-NF training sessions were completed in 1 week. Each session included three runs consisting of eight regulation and nine baseline blocks lasting 30 s each. During the fMRI-NF session, the participants were asked to upregulate the signal using individual mental strategies. However, some template strategies from different cognitive domains were given: positive autobiographic memories, picturing oneself doing sports or playing an instrument, and concentration on given perceptions like feeling the temperature of one’s own left foot. The results demonstrated that both patients with schizophrenia and healthy controls were able to develop control abilities. However, they used different neural strategies: patients activated more of the dorsal and healthy controls activated more of the rostral subdivision of ACC. They also used different mental strategies: patients mainly imagined of music, whereas healthy controls used more imagined sports.

In summary, evidence suggests that patients with schizophrenia are able to learn voluntary control over their brain in spite of their pathology. All of this makes the fMRI-NF a promising tool to tackle frequent and disabling symptoms in this population, such as AVHs.

## What Do we Know about the Neural Basis of Auditory-Verbal Hallucinations?

### Neurocognitive Models

Phonologically, AVHs are heterogeneous in form and content (31). They vary from acousmas (primitive sounds, such as blowing, shooting), utterances, or simple words to full conversations, with defined characteristics such as pitch, volume, and accent. They might consist of a single voice or a collection of voices that speak the individual’s thought aloud, issuing commands and instructions, or provide a running commentary on the person’s behavior. The voices might be familiar or unknown (32). They often carry power, authority (33), and a negative quality [e.g., Ref. (2, 34)], and persons experiencing them often feel that they have no or little control over their AVHs (2).

From a neurocognitive perspective, hallucinations are erroneous perceptions or sensory deceptions without the presence of external stimuli and have been attributed to erroneous integration of sensory and cognitive processes (35) that may influence conscious perception (36). Brain regions that have been implicated in the experience of AVHs include the auditory cortex and the ventral attentional system that spontaneously orientates attention toward an incoming stimulus (37, 38).

A number of neurocognitive models have been proposed to account for heterogenic phenomenology of AVHs (39).^1^ The current models are based on research findings that illustrate the following contributing factors to the experience of AVHs. These are AVHs have clear perceptual qualities, AVHs are internally generated but are not attributed to an internal source, those experiencing AVHs have a reduced sense of control over the onset, content, and frequency of AVHs, and AVHs often carry an emotional component.

Externalization, or lack of agency, was explained by a model proposed by Frith (40), which postulated the breakdown in a physiological process known as self-monitoring. This model is based on the assumption that in patients with schizophrenia, inner speech and/thoughts fail to be recognized as self-generated due to a self-monitoring deficit; reflecting a dysfunction of the efference copy or corollary discharge mechanism that accompanies a motor action, such as speech or movement (41, 42).

In those experiencing hallucinations, the efference copy of inner speech does not produce a corollary discharge of the expected experience. Consequently, this failure in the corollary discharge mechanism can produce confusion regarding the agency between one’s own thoughts and externally generated voice, potentially resulting in an external attribution of the experience and the experience of AVHs. At a neuronal level, this may result in greater activity in the auditory cortex when self-generated speech or inner speech is produced (42, 43).

At a behavioral level, it has been shown that patients with schizophrenia and AVHs exhibit difficulty in identifying self-generated information (44–46). However, models based on the misattribution of inner speech do easily account for observed phenomenology of AVH (47, 48) and there is no evidence that the cancelation or suppression of reafference indicates the source of a sensory event: zero signal is not the same as self-generation (49).

Another early model postulates a deficit in source monitoring or reality testing (50). Source monitoring is a meta-cognitive (thinking about thinking) process that enables us to make attributions as to origins of beliefs and thoughts in order to form a cohesive representation of an experience (50). Bentall et al. suggested that patients with schizophrenia have deficits in discriminating between external (real) and internal (imagined) events, accompanied with a specific externalization bias. For example, it has been demonstrated that patients with schizophrenia and AVHs were more prone to misattribute self-generated items to other sources (51). Further, the experience of AVHs has also been related to deficits in reality testing. Based on signal detection theory (SDT), it was suggested that patients with schizophrenia and AVHs show a shift in the decision criterion (the point at which a person decides they perceive a stimulus) (52). SDT proposes that detection of a stimulus is based on two premises: perceptual sensitivity – the general efficiency of the perceptual system and response bias – the subjective decision criteria to deciding that a perceived event is a stimulus. For example, patients with schizophrenia and AVHs demonstrate higher perceptual sensitivity to detecting words or sounds embedded in white noise, as compared to non-hallucinating patients, but lower sensitivity compared to healthy controls (53). Further, patients with current AVHs also demonstrate a response bias, i.e., indicated that they were certain that a stimulus was presented, even when it was absent, suggesting that the perception/signal detection is unimpaired in patients with AVHs, but there is uncertainty in the signal recognition. This uncertainty, accompanied by a misattribution bias and source/reality monitoring deficits, perpetuates the attribution of thoughts to an external source. This may result in perceptual hypervigilance (54, 55) in responding to biases and lead to (strong) consolidation of such responses with time (56).

Substantial evidence supports the link between AVH and self-, source, and reality monitoring and has been provided over the last two decades (56, 57). Nonetheless, these early models alone cannot account for the presence of AVHs, as they fail to account for certain aspects of their phenomenology. AVHs are often experienced in the second and third person, they may consist of multiple voices that are not the voice of the experiencer, and the experiencers often converse with the AVHs (45, 49).

More recently, a number of models have been developed in order to incorporate the complex phenomenology of AVHs, by integrating the available neurophysiological data and adapting the predictive processing framework (PPF). For example, Allen et al. (35) proposed a neuroanatomical model founded upon a network of brain areas involved in both cognitive and perceptual processing; suggesting that hyperactivation of perceptual regions, including the primary and secondary auditory cortices evident during AVHs (38, 49, 58, 59), and in related speech and language areas (43, 58, 60). On the other hand, Wilkinson (61), adopted the PPF [e.g., Ref. (62)] to account for the phenomenology of AVH. In the framework of PPF, neuronal systems have evolved to predict statistical regularities in the environment based on prior experiences (63). Through successfully encoding predictions in an accurate manner, they minimize prediction errors or deviations from these predictions, and these are seen as the neural systems demonstrating an attenuated response to these predictable events; permitting the serial updating of prediction to create a picture of the external world. This creates a dynamic internal model that can impact on neuronal activity in sensory systems, increasing activity to unpredicted events through a failure of this predictive mechanism, with consequent alterations in subjective perception and elaboration into delusional belief formation (64).

Finally, these recent models suggest a number of cortical and subcortical brain networks involved in the experience of AVH and that verbal hallucinations involve hyperactivity in secondary and primary auditory cortex, accompanied by disrupted coupling with the cognitive processes associated with monitoring/reality testing.

### Neuroimaging Studies

#### Structural Brain Imaging

Structural imaging studies (i.e., studies investigating the brain morphology) have identified subtle but robust reductions in the gray matter volume (GMV) in patients with AVHs, particularly in areas involved in speech and language. Altered GMV in the superior temporal gyrus (STG) has been highlighted by both priori-defined region of interest (ROI) analyses (65) and voxel-based morphometry studies (66). Modinos et al. demonstrated that AVHs severity was significantly associated with GMV reduction in the left STG, including the Heschl’s gyrus. Structural changes have also been identified in Broca’s area and its homotopic contralateral area (67) and the primary auditory cortex (Heschl’s gyrus) (68). In addition to reductions in GMV in language regions, numerous studies have reported modifications in other brain areas, such as temporal and frontal regions (69), insular cortex (70, 71), thalamus (72), and cerebellum (73).

In addition to these quantitative analyses, structural imaging also provides complementary qualitative measures of the cortical morphology, such as the shape of sulci and gyri (74). Indeed, gyrification is considered an indirect marker of brain development since cortical folding (i.e., gyrification and sulcation) begins in the tenth week of gestation and stabilizes by the end of the third trimester of pregnancy. The resulting complex sulcal/gyral patterns are then stable over life (75). Studying changes in cortical morphology associated with AVHs provides a novel way to assess the impact of developmental factors on this symptom (76). Significant reductions in the gyrification of language-related areas (e.g., the superior temporal ridges, the left middle frontal sulcus, Broca’s area) have been identified in chronic schizophrenia patients with AVHs when compared with healthy controls (77). The phenomenology of AVHs has also been associated with morphological changes within the language network. Indeed, the spatial location of AVHs (as internal or external percepts) has been associated with specific sulcal deviations in the right temporoparietal junction (78).

#### Functional Brain Imaging

Functional brain imaging studies in patients with AVHs have provided information about the neural bases of the susceptibility to hallucinate (trait studies), and neural activation that is seen during AVHs (state studies).

##### Trait Studies

Trait studies measure brain activity during specific tasks in patients who hallucinate and those who do not. Inquiring afterward for the absence of AVHs while scanning is necessary to avoid any “state” factor to interfere with this type of paradigm.

Trait studies have revealed altered functional activity in the temporal lobes of patients with AVHs (8, 79). Altered activation is thought to emerge from a competition between AVHs and normal external speech for processing sites within the temporal cortex (80). Similarly designed studies have identified a decrease in the functional activity of the rostral dorsal ACC, a structure known to be involved in the allocation of an internal or external origin for a given stimulus (81, 82). These results are not only compatible with the misattribution models of AVHs (see Neurocognitive Models) but also with recent structural data (83).

##### State Studies

Functional brain imaging suggests that a distributed network of brain regions underlies the experience of AVHs (84). Speech production and comprehension areas have been shown to be involved, but in addition to this network, brain areas involved in contextual memory seem to play a role in AVHs. This was notably revealed by a coordinate-based meta-analysis of AVHs capture studies, which demonstrated increased activity in Broca’s and Wernicke’s areas, and also in the hippocampal complex (84), suggesting that hallucinations could result from the aberrant activation of memory traces within associative cortices (85, 86). Although it is still a subject of debate, the activation of the primary auditory cortex does not appear to be necessary for the occurrence of AVHs. Nonetheless, its activation could be related to specific phenomenological aspects of the hallucinatory experience, such as the feeling of reality (87).

#### Connectivity Studies

Brain connectivity can be studied using three different approaches: functional connectivity, effective connectivity, and structural connectivity (88). Functional connectivity relies on correlation measures between spatially distant brain areas without information on the directionality or causality of the interaction. In contrast, effective connectivity explores the direct influence of one brain region on another, and thus provides information regarding the causal relationship between brain areas in a given network. Finally, structural connectivity is the measure of white matter tracts connecting different brain regions, based on diffusion MRI and tractography algorithms. Many connectivity studies have confirmed the dysconnectivity hypothesis in schizophrenia patients and in particular those who report hallucinations. Indeed, abnormal connectivity between brain regions has been shown at rest [for review, see Ref. (89, 90)] and during verbal tasks by functional and effective connectivity studies (91, 92). This dysconnectivity appears to play a major role in the emergence of hallucinations but was also found to change according to the sensory-modality involved (93, 94). Diffusion MRI studies comparing patients with schizophrenia who experience hallucinations, non-hallucinating patients with schizophrenia, and healthy controls have found differences in the coherence of the white matter bundles connecting language areas (68, 95, 96). This finding was particularly noteworthy in the arcuate fasciculus (97).

## What Strategy to Relieve Auditory-Verbal Hallucinations with fMRI-Neurofeedback?

**Figure 2:**
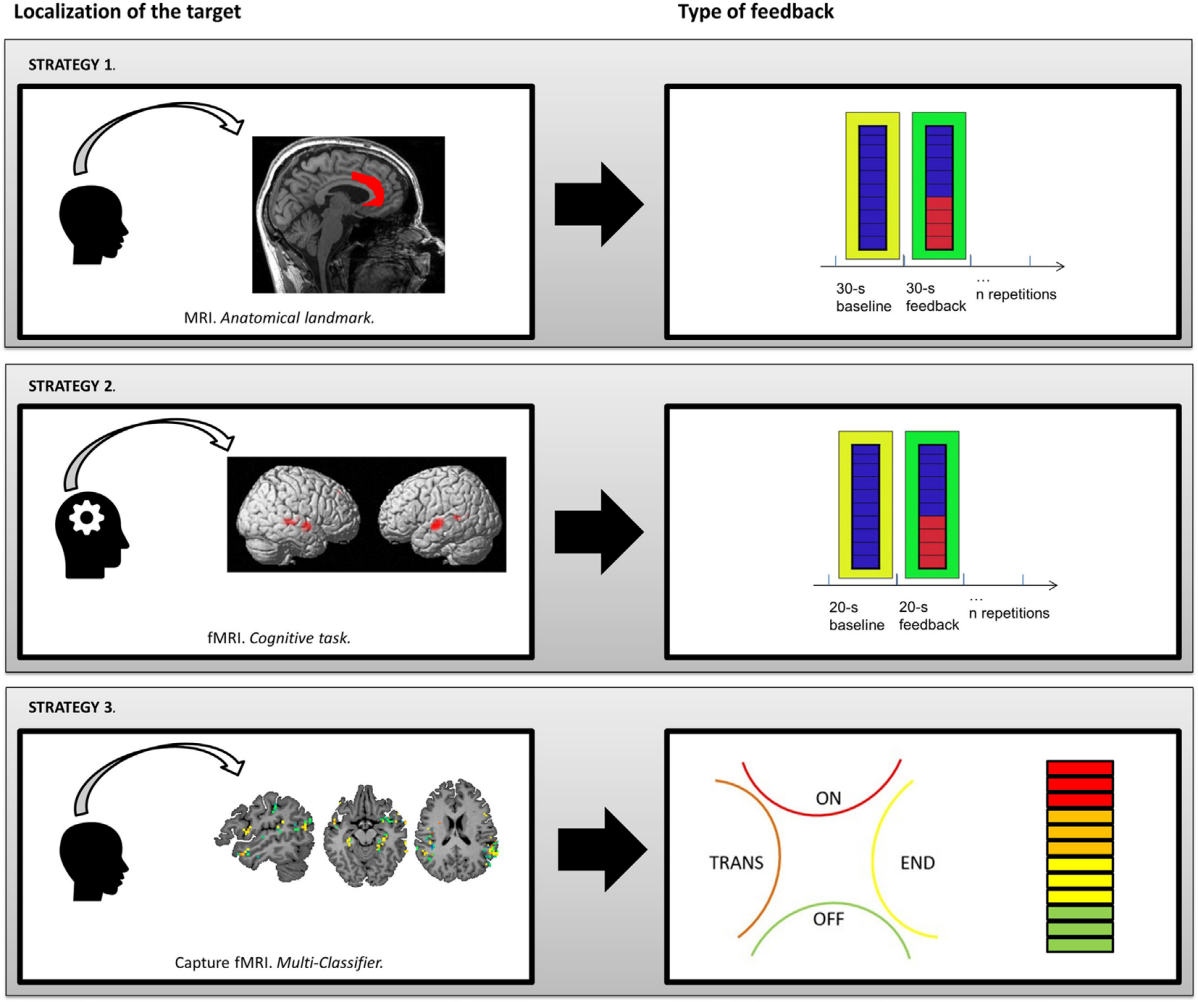
**Presentation of three different strategies for fMRI-neurofeedback protocols to relieve AVHs**. Strategy 1: co-registration of anatomical template of anterior cingulate cortex. Strategy 2: human voice responsive auditory cortex identified with a functional localizer. Strategy 3: linear Support Vector Machine discriminative maps of a classifier after recursive feature elimination steps, which is able to detect neural patterns associated with hallucinations in resting-state brain activity with a level of accuracy of 71%.

In this section, we propose three different fMRI-NF strategies dedicated to AVHs’ treatment on which our teams are currently working on (see Figure 2). We focus on the localization of the target and the type of feedback used for each strategy.

### Strategy 1: *A Priori* Target Localized Using Structural MRI

#### Method Used to Localize the fMRI-Neurofeedback Target

During fMRI-NF protocols, the brain region(s) from which the NF signal is derived can be informed anatomically using structural MRI data and brain atlases (e.g., Talairach and Tournoux coordinates) or according to macroscopic anatomical landmarks. This method is the easiest to implement methodologically but assumes a good understanding of the underlying neural mechanisms and their anatomical location. The goal is to regulate neural activity in areas that show aberrant activations during AVHs (e.g., Broca’s and Wernicke’s areas) or to regulate activity in regions thought to be involved in compensatory or resilience processes (e.g., ACC). Below, we present an fMRI-NF protocol targeting the ACC (see Figure 2, Strategy 1).

#### Why Choose ACC as a Target for fMRI-Neurofeedback to Relieve AVHs?

Disrupted connectivity between the temporal and cingulate cortices has been demonstrated in schizophrenia, with AVHs severity correlating with the connectivity strength between the ACC and the STG (98, 99).

The ACC has a key role in regulating emotions, goal-directed behaviors, attentional processes, response selection, online source monitoring, and cognitive control (100, 101). Moreover, the ACC is involved in differentiating between self- and non-self related stimuli (82, 102). Furthermore, a meta-analysis of trait studies conducted in patients with AVHs and healthy controls revealed decreased ACC activity in hallucinators (79). This finding is in line with cognitive models of AVHs (30, 87).

A number of studies have demonstrated that the ACC can be reliably regulated using fMRI-based NF (13, 17, 103–106). Moreover, the successful upregulation of the rostral ACC was associated with an increase in positive affect (103, 106) and improved emotional perception of voices in healthy subjects (103).

Even though the theoretical accounts differ in the different studies (see Neurocognitive Models), they all assume a failure of typical ACC functions. The monitoring of inner speech processes, the monitoring of retrieval processes and error detection, as well as the suppression of task-irrelevant stimuli are all classical ACC functions that should be fostered by an upregulation of the ACC (107, 108). However, two previous studies report increased ACC activation during hallucinations (58, 109). It is possible that increased ACC activation may be related to default-mode fluctuations, considering simultaneous deactivations of auditory cortex and Wernicke’s area in the former study and resting-state activations without baseline subtraction in the latter study.

#### The fMRI-Neurofeedback Protocol

First, an anatomically predefined ACC mask is applied. From this ROI, the average signal is fed back on a thermometer-like display after filtering and artifact reduction. A custom anatomical template mask of the ACC defines the ROI [details in Ref. (110)]. This ACC mask is taken as a part of the cingulate cortex excluding parts inferior or posterior to the anterior fissure. The feedback signal is the average BOLD signal across this ACC mask for each volume with 1% representing the full scale. A custom toolbox conducts online processing comprising motion correction and co-registration to a template (111). Kalman filter reduces singular values and high-frequency components. An exponential moving average algorithm removes temporal drifts.

The patient performs three fMRI-NF training runs, each consisting of eight regulation blocks and nine baseline blocks (30 s each; see exemplary run in Figure 2). Increase of ACC signal makes a green bar moving upwards and decreasing downwards [Ref. (30)]. A fixed red bar in the regulation condition serves as a regulation target. It indicates the upper limit of ACC upregulation. The baseline condition is indicated by a blue line display. Mental strategies should be tried to move the green line upwards to the red line. During the baseline blocks, the patient counts backwards from 100. Every repetition time (TR; 1 s), the display is updated. The NF procedure is explained to subjects, including the delay of the NF signal for 3–5 s due to the hemodynamic response and data processing (<1 s).

#### Preliminary Data

Patients with schizophrenia can learn to regulate the ACC to a comparable level than healthy controls, albeit involving different networks and cognitive strategies (30). Moreover, a recent article involving three schizophrenia patients suggests that even with ongoing AVHs, patients are able to learn ACC regulation (110). In this work, patients seemed to be very interested in the methodology and were eager to learn. Since the target groups were patients with long-standing symptoms, a core preposition was a good patient–therapist relationship and only limited impairments in cognitive functions. RWTH Aachen University is just performing a clinical trial study investigating the effect of fMRI-NF training in schizophrenia patients with ongoing AVHs.

Previous fMRI-NF studies have demonstrated that upregulation of a single area can elicit alterations of functional and effective connectivity (112, 113). Further studies may elucidate whether ACC upregulation also induces changes of the network dynamics. In the long term, it may be even more effective if fMRI-NF could target several regions aiming to regulate the functional connectivity between these regions. This would allow fMRI-NF to address the neural dysconnectivity that is proposed to underpin AVHs (114). This approach would also enable the regulation of connectivity and activity with the salience network, also proposed to be dysfunctional in people with AVHs (115–117).

### Strategy 2: Region of Interest Defined Using a Functional Localizer

#### Method Used to Localize the fMRI-Neurofeedback Target

The target chosen for fMRI-NF can also be functionally defined. In this case, the patient is asked to undertake a functional task within the scanner, and activated areas are then used as the ROI(s) for fMRI-NF. The choice in the “functional localizer” task should be based on an *a priori* hypothesis that is well validated in previous studies.

#### Why Use a Functional Localizer for fMRI-Neurofeedback to Relieve AVHs?

As already mentioned, in schizophrenia, both state and trait brain imaging studies have revealed aberrant neural activation in patients with AVHs. Resting-state or “non-task” studies suggest that several speech-related areas are linked with such experiences, as well as the ACC and the hippocampal complex (see What Do We Know about the Neural Basis of Auditory-Verbal Hallucinations?). Similarly, task-related paradigms have identified frontotemporal dysconnectivity in patients with AVHs, specifically between the left STG and the dorsal ACC (99) and the medial prefrontal cortex (118), regions thought to be involved in self-other source monitoring. Disruption of these mechanisms is consistent with cognitive models that postulate aberrant bottom-up and top-down processes in AVHs.

Any of these regions could potentially be defined as a target to create a ROI mask for fMRI-NF. However, rather than using a structural or anatomically defined mask, a functional localizer task can be used to define the ROI (119). The choice of an appropriate task for the functional localizer should be informed by previous imaging studies, i.e., studies consistently discriminating the target ROI from other brain activity.

For example, two meta-analyses of AVHs in schizophrenia demonstrated that the human voice sensitive region of the left and right STG is associated with the experience of AVHs (66, 79). Therefore, this region could serve as a potential ROI mask (see Figure 2, Strategy 2). The functional localizer task would need to be designed to specifically identify the human voice responsive auditory cortex [i.e., the task reported in Ref. (120)]. This could be obtained by running of blocks of words (activation) and non-word speech analogs (baseline).

#### The fMRI-Neurofeedback Protocol

After completing data acquisition, the effective signal change measured within the functional localizer tasks is analyzed with univariate fMRI methods, such as the general linear model. The difference between the average BOLD signal of the activation block and the baseline block should be used to create the ROI mask. Several programs offer tools for online analysis, e.g., the AFNI software (http://afni.nimh.nih.gov/afni/). Here, the mask is created by eyeballing the resulting 3D cluster and manually specifying the statistical thresholds until a cluster of the required size/shape is present in the target ROIs. Ideally, the cluster size choice should be informed by previous meta-analytic studies. The mask should also include a control region to serve the averaging out of non-specific brain activation. A randomized controlled trial should also include a control group utilizing a control ROI mask, and each participant should complete both the target and control ROI localizer tasks, in spite of group assignment.

A new mask ROI can be created during each scan, or a retrospective method can be used, whereby the mask obtained during the first visit is used during subsequent neurofeedback trainings. The retrospective method requires the alignment of the different time-series data obtained from different scans. Some MRI scanners allow the realignment of previously obtained data with the current images. However, if this option is not available, most online analysis software have inbuilt algorithms that allow to realigning images obtained during different scanning sessions. The advantage of the retrospective method is the reduction of scanning time and therefore participant discomfort as well as global costs. In addition, the ROI mask does not change shape or size.

In terms of the neurofeedback training, this procedure remains the same as during anatomically masked ROI real-time fMRI, i.e., feedback is provided during the entire training run but remains static during rest (no-regulation blocks). Similarly, participants need to be informed about the inherent delay in feedback due to the hemodynamic response and adhere to standardized instructions. To enhance motivation and the likelihood of successful signal downregulations, participants are instructed to devise their own strategy to downregulate their signal (29, 121).

### Strategy 3: Pattern Recognition Using a Multivariate Classifier

#### Method Used to Localize the fMRI-NF Target

The two previous strategies rely on imaging-based “trait markers” (i.e., persistent traits or vulnerability markers that can also be detected in the presymptomatic and remitted phases of mental disorders). The patient is trained to gain control of areas known to be involved in the AVHs’ pathophysiology. When using such a methodology, the occurrence of hallucinations in the scanner during neurofeedback sessions is not necessary.

However, because hallucinations are acute symptoms, notably characterized by intrusiveness and phasic activity, they can also be targeted with a different type of strategy based on “state markers” (i.e., which correlate with symptomatic states). Here, the objective is to train the subject to self-regulate the activity of brain areas that reactivate during symptomatic states. Machine-learning, and particularly the recent development of “linear Support Vector Machine” (lSVM), offers several advantages in this context. Indeed, this technique classifies functional or anatomical patterns using a multivariate strategy. A training session allows the optimal classifier to be built on the basis of a training dataset, for which the periods of interest (e.g., symptomatic vs. asymptomatic) have been identified and provided (122). A validation session is then needed to test the performance and possible generalization of this classifier to new data based on an independent sample. Several interesting results for diagnosis or therapeutic response prediction purposes have been published, notably in bipolar disorder (123) or schizophrenia (124). However, this is not the only way to use such tools in psychiatry. Classifiers can quickly detect the emergence of subjective symptoms by detecting specific patterns of brain activity identified during symptomatic periods (see Figure 2, Strategy 3).

#### Why Use Classifiers for fMRI-NF to Relieve AVHs?

Using fMRI classifiers, it is now possible to detect the onset of subjective symptoms together with the associated brain activation patterns (125, 126). For example, our group developed such a classifier to detect AVHs occurrence while scanning a patient with a 71% accuracy (127). This algorithm is currently under optimization and already reaches 80% accuracy. Even if no data are currently available on the use of this kind of classifier in fMRI-NF protocols, the fine-grained activity patterns obtained could theoretically be used as the signal fed back to the patient. Future studies should allow specifying the minimal necessary accuracy.

However, to be eligible for this strategy, the patient’s hallucinations must exhibit some specific features. The most important criterion is frequent occurrence. Indeed, the symptom must occur several times during the fMRI session. Moreover, data analysis and patient interviews must allow the identification of “symptomatic” and “asymptomatic” periods to build an efficient classifier. In our case, we chose to build a subject-independent classifier based on the AVHs presence or absence, determined with the methodology described in Ref. (87). This strategy presents substantial benefits compared with a subject-dependent pattern classification of fMRI signals, notably a considerable time-saving (128).

#### The fMRI-Neurofeedback Protocol

Unlike the two methods described above, this strategy does not imply a block paradigm. Indeed, the visual feedback provides an information in real time about the current state of the participant (hallucinating or not) all along the session. The visual feedback may be a thermometer whose signal intensity is based on the level of activation in the ROIs (given by the discriminative maps of the classifier). But, other possibilities emerged from recent work on AVHs. Our team recently proposed a method to distinguish between the different periods in the occurrence of AVHs (117). Even if we are at a very preliminary stage, this could theoretically allow for the implementation of a multi-classifier strategy with the possibility to discriminate multiple “brain-states” as, in our case, (i) “No hallucination” (“Off” period on Figure 2), (ii) “Transition” (“Trans” period on Figure 2; i.e., period immediately preceding the AVHs occurrence), (iii) “Hallucination” (“On” period on Figure 2), and (iv) “End” (“End” period on Figure 2; i.e., period immediately following the AVHs occurrence). This technique can provide a feedback indicating which “brain-state” is identified. For example (as presented in Figure 2), a four-part diagram presenting the four brain-states can be used. If the “hallucination” period or “transition” period is identified, the participant must adapt his/her mental strategy to go back to “end” or “no hallucination” periods. This kind of feedback could also be combined with a thermometer display (to provide both a continuous and a discrete variable to the subject).

## Limits and Future Directions

### fMRI-Neurofeedback Experimental Designs

The most obvious limitation of the available studies testing fMRI-NF protocols are their small sample sizes, making generalization difficult. For AVHs, no study assessing the efficacy of fMRI-NF is currently available. Nevertheless, the improved understanding of the neural underpinnings of AVHs seen in recent years and the preliminary results presented here should inform future studies.

The gold-standard to assess new treatments is the double-blind, randomized controlled trial design. However, a major issue in fMRI-NF protocols is to achieve complete “blindness” in patients, because an active collaboration is needed during the sessions. This directly questions what could be an ideal control condition? Four kinds of control conditions have been described in the literature (11) (i) mental task outside of the scanner, (ii) sham feedback using brain signal of interest from previous participant, (iii) sham feedback using inverse brain signal of interest, and (iv) sham feedback using brain signal from an unrelated region. The first solution appears unsatisfactory because patients in the control group are not exposed to fMRI-NF. Using a brain signal of interest from previous participants may generate frustration and retention since participants may unravel the non-contingency of the feedback, which would unblind them and reduce their engagement with the intervention. Moreover, for patients with severe AVHs, this kind of feedback could increase anxiety, letting them think that they have no control on their neural activity. Using an inverse brain signal of interest is unethical in the specific case of AVHs treatment. Indeed, this kind of sham feedback aims to test if inverse brain modulation prompts opposite behavioral changes. As a consequence, the expected change would be a worsening of AVHs symptomatology. Neurofeedback from a non-interest region should be the “least bad” solution for a control condition in fMRI-NF protocols to treat AVHs. The selection of a non-interest region appears crucial here and could be a difficult challenge, given the complexity (and spread) of the brain networks involved in AVHs (unfortunately, no data are currently available on the potential non-interest ROI that could be used for protocol, testing the efficiency of fMRI-NF in AVHs).

Another significant challenge to adequately assess neurofeedback effectiveness is to develop dedicated post-session scales that are able to identify the specific cognitive coping strategies used by the patients during the session. Such individualized strategies could then be applied in psychotherapy, potentially leading to the development of neuroimaging-guided programs. A rigorous evaluation of the strategies used to cope with AVHs during the fMRI-NF sessions could then be helpful to optimize general hallucination-focused psychotherapy programs. We believe that this may constitute an interesting two-way relationship between conventional psychotherapy and fMRI-NF: fMRI-NF is a precious tool to optimize hallucination-focused psychotherapy programs, while the identification of brain changes after psychotherapy allows for the identification of new neurofeedback targets.

Finally, testing whether brain self-regulation persists after the fMRI-NF protocols is a crucial issue. The “transfer session” (see Figure 1) may provide information about the capacity of participants to self-regulate the target region(s) without feedback. Furthermore, it will be very important to determine how long this capacity persists after the fMRI-NF and how long the clinical improvement is maintained. To date, no formal follow-ups of symptoms were conducted with the patients. The question of the potential long-term effects of these treatments is clearly under-assessed in *fMRI Brain–Computer Interface* research in general (113) and no data are currently available for patients with schizophrenia.

### fMRI-Neurofeedback Protocols

In addition to the non-invasive nature of fMRI-NF, one of its prominent features is to put the patient at the heart of the process. On one hand, the active participation of the patient in fMRI-NF may contribute to the reinforcement of their feeling self-efficacy [which constitutes an important therapeutic factor (129)]. On the other hand, this active nature may be source of limitations in schizophrenia patients with strong negative symptoms, who may lack motivation. Although some data seem to indicate that patients suffering from schizophrenia are able to achieve voluntary control of their own brain activity during fMRI-NF (29, 30), these results need to be confirmed in studies with larger samples. Given the importance of motivation in neurofeedback protocols, it seems very relevant to consider factors interfering with reward processing, such as negative symptoms and antipsychotic medication. The effort required by patients to undergo the fMRI-NF training should not be underestimated as well as the mixed motivation of the patients, since there often exists some positive aspects to the hallucinatory experiences, which the patients may fear losing as a result of the training. Future research will have to determine what the best experimental settings/instructions are for patients suffering from refractory AVHs together with severe negative symptoms.

Considering task design, the most of fMRI-NF studies use a block design (i.e., alternating periods of upregulation or downregulation with rest periods during neurofeedback runs, see Figure 1). However, the optimal number of blocks per run, the ideal duration of regulation blocks, and the best number of sessions to obtain a maximal efficacy in the treatment of AVHs remain unknown. Future research should determine if patients suffering from schizophrenia (particularly those who exhibit severe cognitive impairment) may benefit from special arrangements in fMRI-NF protocols to minimize the attention span and the tiredness.

Informal reassessments during clinical visits that were conducted during the pilot study of the currently ongoing study with schizophrenia patients with AVHs (see above) revealed that none of the patients reported adverse events, and two of the patients claimed to have developed different strategies in dealing with their AVHs up to few weeks after the training (110). However, during contact and assessment occurring more than a month after the training, none of the patients had the impression that fMRI-NF training had had any influence on their symptoms. Individual variability and fluctuation in the disease course may override the – so far rather small – effects of the fMRI-NF training. This may change with better targeted fMRI-NF protocols. However, based on clinical impressions, we would suggest that at least monthly booster session would be advisable for clinical trials.

From a methodological point of view, uncertainty lies also about the instructions to be given before the session. It remains unknown if explicit (the participant is asked to use specific mental strategies for self-regulation) or implicit (the participant is only asked to upregulate or downregulate with the feedback provided) instructions should be preferred. Implicit instructions are ideal in general population, because they favor the development of individualized strategies to achieve voluntary control of the target region(s). However, the identification of an optimal strategy may be difficult for patients with severe AVHs, which could lead to a rapid decline in motivation. That is why providing specific explicit strategies could be useful to enhance the efficacy of fMRI-NF to treat AVHs. Strategies inspired from CBT could allow the participant for achieving voluntary control more quickly.

Finally, considering the definition of the fMRI-NF target, many other neural networks may serve as target for the fMRI-NF training. Interestingly, one of the most robust effects of fMRI-NF training seems to be changes in connectivity [e.g., Ref. (112, 130)]. Indeed, the first fMRI-NF studies attempt to train network connectivity directly (131, 132). Considering the importance of network function on the AVHs phenotype, connectivity fMRI-NF will be one of the next targets for treatment approaches to AVHs.

## Conclusion

Although a number of studies are currently investigating the efficacy of fMRI-NF for AVH, as for today, efficiency data from randomized controlled trials are lacking (11). In this paper, we focused on specific fMRI-NF strategies to treat AVHs and selected three of them that appear most feasible, emphasizing the need for preliminary studies. Indeed, considering the potential cost necessary to implement fMRI-NF, proof-of-concept studies are urgently required to define the optimal strategy for application in patients with AVHs. This technique has the potential to establish a new brain imaging-guided psychotherapy for patients that do not respond to conventional treatments and take functional neuroimaging to therapeutic applications.

## Author Contributions

TF, NO, MD, PA, KM, and RJ equally contributed to the manuscript writing.

## Conflict of Interest Statement

The authors declare that the research was conducted in the absence of any commercial or financial relationships that could be construed as a potential conflict of interest.

## Footnotes

^1^Belzeaux R, Cermolacce M, Jardri R. Hallucinations: toward a dialogue between phenomenology and brain imaging research. *J Conscious Stud* (Forthcoming).
